# *PCDH17* gene promoter methylation status in a cohort of Egyptian women with epithelial ovarian cancer

**DOI:** 10.1186/s12885-023-10549-3

**Published:** 2023-01-25

**Authors:** Sherif Mohamed Elsharkawi, Dalal Elkaffash, Pacint Moez, Nour El-Etreby, Eman Sheta, Raghda Saad Zaghloul Taleb

**Affiliations:** 1 Department of Clinical and Chemical Pathology, Faculty of Medicine, Alexandria University, Alexandria, Egypt; 2 Department of Obstetrics and Gynecology, Faculty of Medicine, Alexandria University, Alexandria, Egypt; 3 Department of Pathology, Faculty of Medicine, Alexandria University, Alexandria, Egypt

**Keywords:** Ovarian cancer, *PCDH17*, Pyrosequencing, Epigenetics, DNA methylation

## Abstract

**Background and objective:**

Ovarian cancer is a leading cause of female mortality. Epigenetic changes occur in early stages of carcinogenesis and represent a marker for cancer diagnosis. Protocadherin 17 (*PCDH17*) is a tumor suppressor gene involved in cell adhesion and apoptosis. The methylation of *PCDH17* gene promoter has been described in several cancers including ovarian cancer. The aim of the study was to compare the methylation status of *PCDH17* gene promoter between females diagnosed with epithelial ovarian cancer and a control group composed of normal and benign ovarian lesions.

**Methods:**

Fifty female subjects were included in our study (25 ovarian cancer patients and 25 controls). DNA was extracted from Formalin-Fixed Paraffin-Embedded (FFPE) tissues of the subjects. Methylation levels for six CpG sites in the *PCDH17* gene promoter were assessed by pyrosequencing.

**Results:**

The methylation levels at five out of six sites were significantly higher in females with epithelial ovarian cancer compared to the control group. Moreover, the same applies for the mean methylation level with *p* value 0.018.

**Conclusion:**

Methylation of *PCDH17* gene promoter plays a role in ovarian carcinogenesis and can be used for diagnosis and early detection.

## Introduction

Worldwide, there are about 314,000 new ovarian cancer cases per year. Ovarian cancer represents the 9th cancer in order of frequency among cancers affecting females. It is the fourth most common cause of cancer-related deaths in women accounting for 204,252 deaths per year [1, 2]. The reason for this relatively increased mortality rate is the late diagnosis of patients as in early stages they are asymptomatic or present with vague symptoms [3, 4]. The majority of ovarian cancer cases are of epithelial origin; epithelial ovarian cancer. About 75% of these cases are diagnosed at stages IIIc and IV where the 5-year survival rate is less than 30% [5].

CA125, a marker for ovarian cancer, lacks the desired sensitivity and specificity to allow for confident screening and diagnosis. CA125 level fluctuations occur due to some physiological factors. Non-neoplastic disorders, benign ovarian tumors as well as non-ovarian malignancies are all associated with an increase in CA125 levels. CA125 correlates with tumor burden and hence its sensitivity is reduced in early stages of the disease [6].

Human epididymis secretory protein 4 (HE4) has better performance than CA125. However, it does not provide a useful tool for early diagnosis [7]. CA125 and HE4 are mainly approved for monitoring treatment and detecting recurrence [8].

Multiple panels have been proposed for diagnosis of ovarian cancer: risk of malignancy index (RMI), risk of ovarian malignancy algorithm (ROMA), Copenhagen index (CPH-I), OVA1® and OVERA®. They combine various laboratory markers in addition to other data (e.g., clinical, radiological). They have better performance compared to single markers. They are used as triage tests or referral tests and evaluating adnexal masses [7–9]. However, they do not achieve the required sensitivities and specificities to be used for screening.

Epigenetics implies changes in nucleic acids (and histones) that are not related to DNA sequence (e.g., DNA methylation and histone modification). During carcinogenesis, several genetic and epigenetic alterations occur simultaneously [10, 11]. DNA methylation occurs via the chemical addition of a methyl group to the 5′- carbon of cytosine residues in CpG islands. CpG islands are found in gene promoters near to transcription start sites (TSS) and mostly unmethylated whereas 5-methylcytosines, not present in CpG islands, are frequently methylated and undergo deamination to thymine [12]. Methylation of CpG islands is often associated with silencing and decreased expression of the respective gene [13, 14]. DNA hypermethylation of the promoters of tumor suppressor genes results in their silencing and favors, along with other mechanism, carcinogenesis [12, 15]. DNA hypermethylation occurs early in ovarian cancer and represents a potential screening target [16].

Protocadherins (*PCDHs*) are a part of the cadherin family and are considered to be of the tumor-suppressor genes. *PCDHs* are divided into clustered and non-clustered *PCDHs*. Clustered *PCDHs* include α, β and γ groups; whereas non-clustered *PCDHs* include δ1, δ2 and ε groups. One of the δ2 *PCDHs* is protocadherin 17 (*PCDH17*) [17]. Like other *PCDHs*, *PCDH17* is a tumor suppressor gene and its promoter shows hypermethylation in different cancers (e.g., breast, prostate) [18, 19]. *PCDH17* promoter hypermethylation has been reported in ovarian cancer [20–22]. Therefore, the purpose of this study was to assess the methylation status of *PCDH17* gene promoter in epithelial ovarian cancer patients.

## Methods

### Study participants

We performed sample size calculation for our study using G.power 3.1.9.2 (Kiel University, Germany). The sample size was calculated according to the role of *PCDH17* gene promoter hypermethylation described by Baranova and colleagues [20]. Based on the following considerations: 0.05 α error, 85% power of the study and allocation ratio of 1:1. Two cases were added to overcome drop-out. The conclusion was to allocate 25 subjects in each group.

A total of 50 subjects were included in this study who underwent oophorectomy (separately or as a part of a more radical surgical procedure) between August 2020 and November 2021 at the department of gynecological oncology in Elshatby University Hospital, Alexandria, Egypt. They were divided into two groups: (A) Control group: 25 female subjects that underwent oophorectomy with benign ovarian masses or normal ovarian tissue and (B) Patient group: 25 female subjects who have been recently diagnosed with epithelial ovarian cancer. Diagnosis was done post-operatively and histopathological examination of the surgical specimen. Patients who have received chemotherapy or who have been diagnosed with other malignancy were excluded. A written informed consent was obtained from each subject enrolled in this study.

Before surgery, full history was obtained regarding age, pregnancy, parity, lactation, menopausal status, intake of oral contraceptive pills (OCPs) and complaint(s)/symptoms. Family history regarding ovarian cancer was addressed also. Pelvic ultrasonography was performed. In the case of presence of adnexal mass, the following elements were assessed (mass measurement, unilateral mass versus bilateral masses, presence of solid areas, presence of multilocular cysts, evidence of metastases and ascites) and used to calculate risk of malignancy index (RMI).

### Sample collection

Blood was obtained from all subjects by sampling from the antecubital veins. Serum was stored at -20 °C. After histopathological examination and demarcating the block containing the tumor tissue (in benign or malignant cases) or containing the normal ovarian tissue (in normal cases), formalin-fixed paraffin-embedded (FFPE) tissue was obtained for each subject. For each paraffin block, 3 sections were discarded and then 8 sections of 5-10 μm thickness were cut using a scalpel and transferred to a sterile eppendorf tube.

### Laboratory investigations

CA125 was measured using Cobas e411 (Roche Diagnostics, Switzerland).

### Detection of methylation status of PCDH17 gene

#### Genomic DNA extraction

DNA extraction was performed using QIAamp® DNA FFPE tissue kit (Qiagen, Germany, Cat no: 56404) following manufacturer’s instructions. The purity and concentration of DNA was assessed using the NanoDrop™ 2000 spectrophotometer (ThermoFisher Scientific, USA). DNA was then stored at -20 °C till further analysis.

### Bisulfite treatment

Bisulfite treatment was done using EpiTect® Fast DNA Bisulfite Kit (Qiagen, Germany, Cat no: 59824) adhering to manufacturer’s instructions. Bisulfite treatment converts any unmethylated cytosines to uracil while methylated cytosine residues remain the same. Converted DNA was then stored at -20 °C till further analysis.

### DNA amplification

The converted DNA was amplified using PyroMark® PCR Kit (Qiagen, Germany, Cat no: 978703) and SimpliAmp Thermal cycler (ThermoFisher Scientific, USA). Amplification primers were obtained from Qiagen, Germany (Hs_PCDH17_02_PM PyroMark® CpG Assay, Cat no: 978746). The sequence is CCCGACTTGCTGCGCCCTCCGCCGCCGCGC. PCR was done following manufacturer’s instructions. The initial DNA concentration was 200 ng/reaction. The amplification protocol consisted of an initial denaturation step at 95^o^ C for 15 minutes, 45 cycles of denaturation at 94 °C for 30 seconds, annealing at 56 °C for 30 seconds and extension at 72 °C for 30 seconds followed by a final extension step at 72 °C for 10 minutes. The amplification products (247 bp) were separated by 1% agarose gel electrophoresis and ethidium bromide dye. Products were then viewed by transillumination using a UV scanner.

### Pyrosequencing

Sequencing was performed using PyroMark® Q24 instrument (Qiagen, Germany). The reagents used included the sequencing primer of the CpG Assay (cat no. 978746), PyroMark® Gold Q24 reagents (Qiagen, Germany, Cat no 970802), PyroMark® Binding Buffer, PyroMark® Denaturation solution, PyroMark® Wash Buffer and PyroMark® Annealing Buffer. The sequence of the sequencing primer is depicted above.

The mixture reaction used composed of 1 μl streptavidin-coated sepharose beads, 40 μl Binding Buffer, 15 μl PCR product and 24 μl DNase/RNase-free water (total volume 80 μl). Preparing the PyroMark® workstation was done with freshly prepared 70% ethanol, denaturation solution, wash Buffer and ultrapure water. Annealing Buffer (22.5 μl) and sequencing primer (2.5 μl) were placed in each well of the PyroMark® Q24 plate. In addition, the enzyme, substrate mix and nucleotides (of the Gold Q24 reagent) were placed in their respective places of the PyroMark® Q24 cartridge which was then transferred to the device.

Immediately after DNA immobilization onto sepharose beads, PCR strips and the PyroMark® plate were transferred to the PyroMark® Q24 Workstation. Beads were captured by the vacuum tool of the workstation and then washed in ethanol. Denaturation to prepare single-stranded DNA was done by the Denaturation Buffer. After wash with ultrapure water, beads were released into the wells of the plate. The plate, containing the samples, was then heated to 90 °C and then allowed to cool for 5 minutes to allow annealing of the single stranded DNA to the sequencing primer. The plate was then transferred to the PyroMark® Q24 pyrosequencing was initiated.

Methylation was assessed using PyroMark Q24 2.0.8 Build 3 software (Qiagen, Germany). Methylation was calculated for each site of the six CpG sites included in the assay. The percentage of methylation was calculated as follows: Percent of cytosines (methylated)/ percent of cytosine and thymine (methylated and unmethylated) [23].

### Statistical analysis

Statistical analysis was performed using IBM SPSS software package v20.0 (Armonk, NY, IBM Corp). Qualitative data were described using number and percentage. Categorical variables were analyzed using Chi-square test / Fisher’s exact or Monte-Carlo correlation. For quantitative data, the Shapiro-Wilk test was used to assess normality of distribution. Student t-test was used to compare between the two studied groups regarding normally distributed data. Non-normally distributed data were compared using Mann Whitney test or Kruskall Wallis test. The accepted level of significance was stated at 0.05 (*P* value ≤0.05 was considered significant).

The methylation of each site of CpG sites were compared between the two studied groups in addition to the mean % of methylation.

## Results

**Fig. 1:**
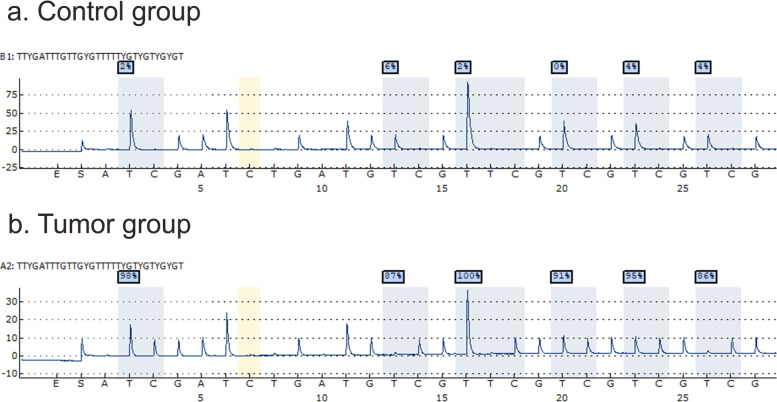
Pyrogram produced by pyrosequencing of *PCDH17* gene promoter. Above; control group. Below; Tumor group. Six CpG sites are demarcated by blue highlights (1 to 6, left to right). The value above each site represents the % of methylation of this site in an individual patient. The yellow color represents the “bisulfite conversion control”. Dispensation order lies on the abscissa and relative light intensity lies on the ordinate

Fifty subjects were enrolled in this study; half of them represents the control group and the other half represents the cancer group. Methylation status for each one of the six CpG sites included in the PyroMark® Assay were performed and analyzed using PyroMark® Q24 software. Figure 1 shows the pyrogram for one case and one control subject.

Subjects were compared in relation to their demographic, clinical, radiological, laboratory and pathologic data as shown in Tables 1 and 2. No family history for ovarian cancer was recorded for all subjects.

**Table 1:** Different demographic, clinical and laboratory data between the two studied groups

Parameter	Control group	Patient group	***P*** value
**Age (years)**			
Mean ± SD	49.08 ± 10.9	45.88 ± 13.4	0.359
**Pregnancy**			
Median (IQR)	3 (3-4)	2 (0-3)	0.015*
**Parity**			
Median (IQR)	3 (3-4)	2.12 (0-3)	0.025*
**Lactation**	22/25	14/25	0.012*
**Menopause**	9/25	6/25	0.355
**Tubal ligation**	0/25	1/25	0.49
**Oral Contraceptive Pills**	1/25	2/25	1
**Complaints/symptoms**			
Abdominal pain	14/25	19/25	0.136
Distension	5/25	13/25	0.018*
Bleeding	13/25	2/25	0.001*
Constipation	1/25	1/25	FEp = 1.0
Weight loss	0/25	2/25	FEp = 0.49
Discharge	0/25	1/25	FEp = 1
Heaviness	1/25	0/25	FEp = 1
Oligomenorrhea	0/25	1/25	FEp = 1
Amenorrhea	0/25	1/25	FEp = 1
**CA125 (U/ml)**			
Median (IQR)	15.8 (13.7-31.3)	175 (68.7-766)	< 0.001*

*FE* Fischer Exact Probability, *p p* value, *SD* standard deviation, *IQR* interquartile range

*P value is statistically significant at *p* ≤ 0.05

**Table 2:** Comparison between control group with adnexal masses and malignant group

Parameter	Control with adnexal mass group	Malignant group	*P*
**Ultrasound feature**			
Mass size (greatest dimension in cm)			
Mean ± SD	14.53 ± 7.85	13.06 ± 5.68	0.496
**Nature**			MCp = 0.001*
Solid	14	10	
Cystic	1	2	
Mixed	0	12	
Bilateral with different nature	0	1	
**Solid areas**	3/12	21/25	< 0.001*
**Bilateral masses**	1/15	7/25	FEp = 0.219
**Multilocular cysts**	10/12	17/25	FEp = 1
**Metastases**	0/12	1/25	FEp = 1
**Ascites**	0/15	4/25	FEp = 0.278
**CA125**			
Median (IQR)	15.4 (14.3-66.15)	175 (68.7-766)	< 0.001*
**RMI**			
Median (IQR)	30.2 (15.95-75.9)	370.8 (91.8-1092)	< 0.001*

*MCp* Monte Carlo Correlation, *FE* Fischer Exact Probability, *p p* value, *SD* standard deviation, *IQR* interquartile range, *RMI* Risk of Malignancy Index

*P value is statistically significant at *p* ≤ 0.05

Out of the 25 control subjects, 10 did not present with adnexal mass and ovaries were unremarkable in pathology. The other 15 subjects were diagnosed as follows: unremarkable ovaries (*n* = 2), ovarian cyst (*n* = 1), mucinous cystadenoma (*n* = 7), serous cystadenoma (*n* = 3) and cystadenoafibroma (*n* = 2). For the 25 tumor subjects (epithelial ovarian cancer), they were diagnosed as follows: serous carcinoma (*n* = 12), mucinous carcinoma (*n* = 4), endometrioid carcinoma (*n* = 4), mixed epithelial carcinoma (*n* = 2), malignant Brenner (*n* = 1) and epithelial carcinoma with the exact type cannot be determined (*n* = 2). The staging of malignancy was performed in 18 subjects; stage I (*n* = 9), stage 2 (*n* = 6) and stage 3 (*n* = 3).

**Fig. 2:**
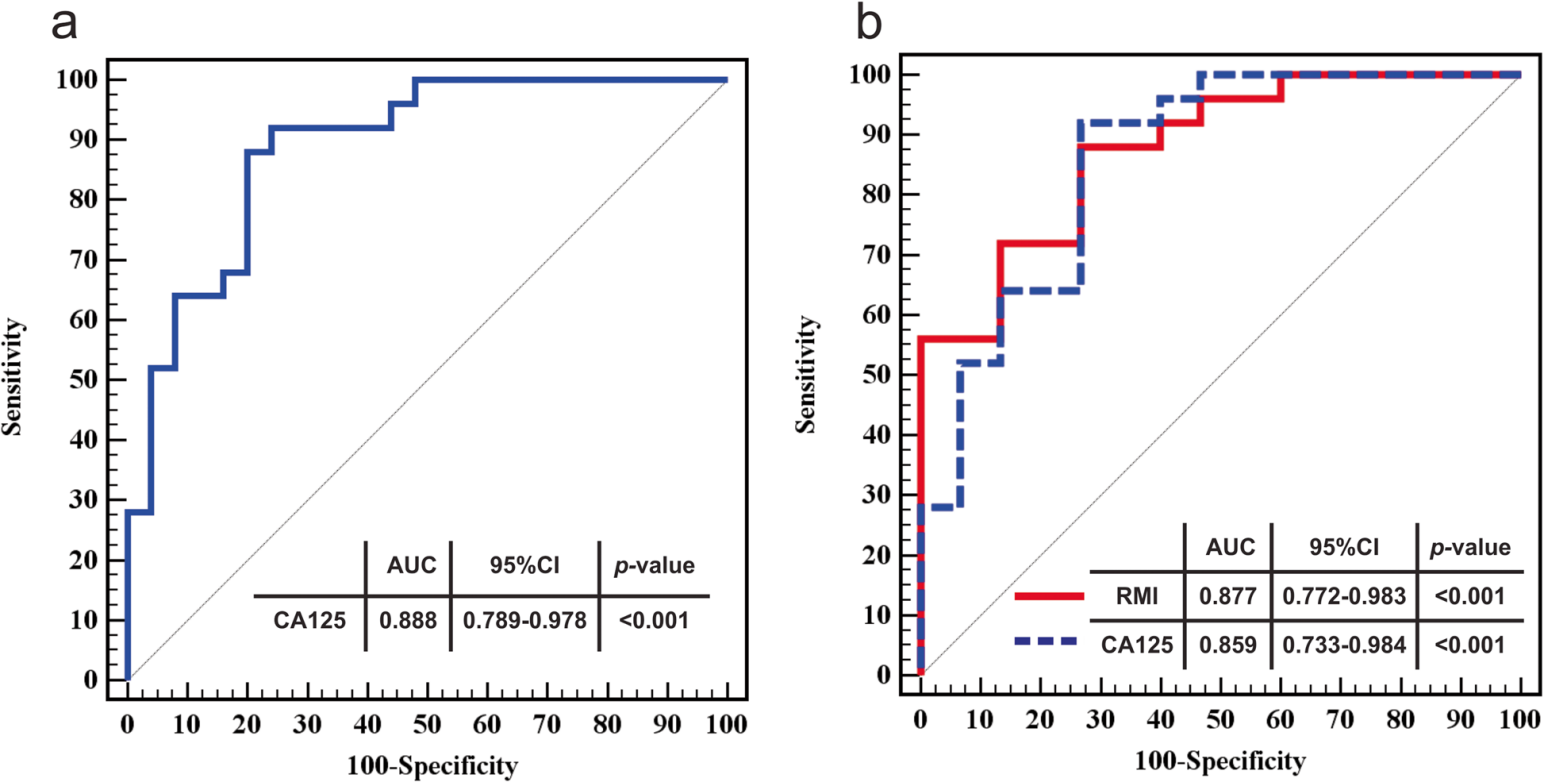
ROC curves for CA125 and RMI. **a**. ROC curve for CA125 between all control subjects and malignant group **b**. ROC curve for CA125 (blue) and RMI (red) for control subjects with adnexal masses and malignant subjects

CA125 was found to be have a statistically significant difference between both groups (Table 1). Similarly, CA125 and RMI was statistically significant between cases and control group subjects presenting with ovarian masses (Table 2). ROC curve analysis (Fig. 2) was performed as well as sensitivity, specificity, positive predictive value (PPV) and negative predictive value (NPV). When using CA125 to discriminate between control subjects and patients with ovarian cancer (cut-off 33 IU/ml), area under the curve (AUC) was 0.888 (95% C.I. 0.798-0.978), sensitivity was 88%, specificity was 76%, PPV was 78.6% and NPV was 86.4%. When using CA125 to discriminate between control subjects with masses and patients with ovarian cancer (cut-off 33 IU/ml), AUC was 0.859 (95% C.I. 0.733-0.984), sensitivity was 88%, specificity was 73.33%, PPV was 84.6% and NPV was 78.6%. Regarding the diagnostic performance of RMI to differentiate between the latter groups, AUC was 0.877 (95% C.I. 0.772-0.983), sensitivity was 88%, specificity was 73.33%, PPV was 84.6% and NPV was 78.6%.

**Fig. 3:**
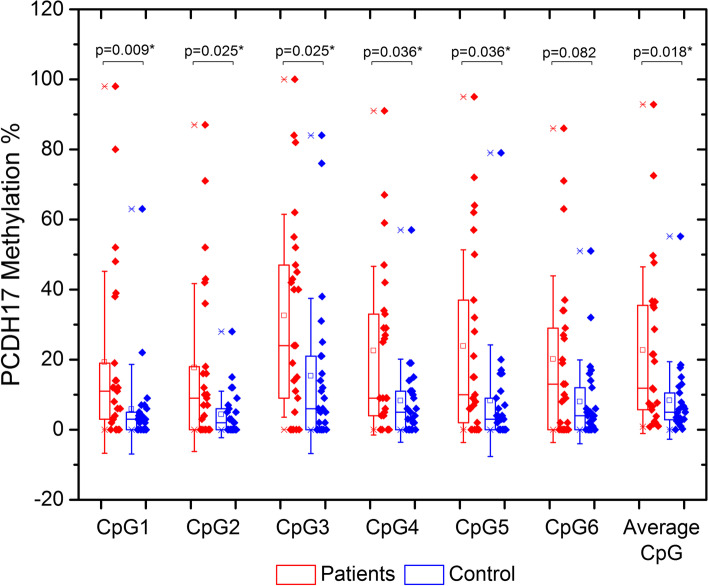
Box and Whisker plot for methylation profiles of the 6 CpG sites and mean methylation in both studied groups. Boxes represent 25th and 75th percentiles. Whiskers indicate the 1.0 fold standard deviation. The median is shown as a horizontal line and the mean value as a square within the box. Individual data points are displayed as filled diamonds beside the boxes. Patients are represented by red diamonds while control subjects are represented by blue diamonds. *P* values are shown above the plots for each site. Significant differences between patients and controls are indicated by an asterisk

*PCDH17* promoter methylation levels were found to be different between both study groups. Statistically-significant hypermethylation were found in the first 5 out of 6 CpG sites (*p* values 0.009, 0.025, 0.025, 0.036 and 0.036) and in site 6, cases were hypermethylated but no statistical significance was found (*p* value = 0.082). When comparing the mean CpG methylation values for the six sites in the assay, the difference was statistically significant (p 0.018). In addition, we compared the methylation status between both subgroups of the control (with and without mass) and the malignant groups. These results are shown in Table 3. Furthermore, we investigated the methylation percentages between normal and benign control which were found to show no statistical difference. These results are shown in Table 4. Figure 3 shows the Whisker-Box distribution of methylation values in the 6 CpG sites among control and malignant groups.

**Table 3:** Methylation percentages between the studied groups in the six CpG sites

CpG site	Control group		Malignant group	*P*		
**CpG 1**				0.009*		
Median (IQR)	3 (0-5)		11 (3-19)			
**CpG 2**				0.025*		
Median (IQR)	2 (0-6)		9 (0-18)			
**CpG 3**				0.025*		
Median (IQR)	6 (0-21)		24 (9-47)			
**CpG 4**				0.036*		
Median (IQR)	5 (0-11)		9 (4-33)			
**CpG 5**				0.036*		
Median (IQR)	3 (0-9)		10 (2-37)			
**CpG 6**				0.082		
Median (IQR)	4 (0-12)		13 (0-29)			
**Average CpG**				0.018*		
Median (IQR)	5 (2.83-10.5)		11.83 (5.67-35.5)			
**CpG site**	**a) Control without mass**	**b) Control with mass**	**c) Malignant group**	**P1**	**P2**	**P3**
**CpG 1**				0.016^*^	0.255	0.004^*^
Median (IQR)	3.50 (3.0 – 7.0)	0.0 (0.0 – 4.0)	11 (3-19)			
**CpG 2**				0.078	> 0.05	> 0.05
Median (IQR)	2.50 (0.0 – 6.0)	0.0 (0.0 – 6.0)	9 (0-18)			
**CpG 3**				0.055	> 0.05	> 0.05
Median (IQR)	5.50 (0.0 – 12.0)	11.0 (1.0 – 23.0)	24 (9-47)			
**CpG 4**				0.044^*^	0.013^*^	0.253
Median (IQR)	1.50 (0.0 – 11.0)	6.0 (3.0 – 12.50)	9 (4-33)			
**CpG 5**				0.074	> 0.05	> 0.05
Median (IQR)	3.0 (0.0 – 6.0)	4.0 (1.0 – 13.5)	10 (2-37)			
**CpG 6**				0.075	> 0.05	> 0.05
Median (IQR)	2.0 (0.0 – 5.0)	6.0 (1.50 – 16.5)	13 (0-29)			
**Average CpG**				0.036^*^	0.014^*^	0.123
Median (IQR)	3.17 (2.67 – 6.67)	5.83 (3.17 – 12.67)	11.83 (5.67-35.5)			

*p p* value, *IQR* interquartile range, *P1 p* value between groups a,b and c. *P2 p* value between groups a and c. *P3 p* value between groups b and c

*P value is statistically significant at *p* ≤ 0.05

**Table 4:** Methylation percentages between the normal and benign control subgroups in the six CpG sites

CpG site	Normal control	Benign control	*P*
**CpG 1**			0.574
Median (IQR)	3 (2-4)	1.5 (0-5.5)	
**CpG 2**			0.728
Median (IQR)	2 (0-3)	2.5 (0-8)	
**CpG 3**			0.81
Median (IQR)	6 (2-12)	10 (0-23)	
**CpG 4**			0.137
Median (IQR)	3 (0-9)	8 (3-14.5)	
**CpG 5**			0.437
Median (IQR)	3 (0-6)	4 (1-13.5)	
**CpG 6**			0.11
Median (IQR)	3 (0-5)	6 (1.5-16.5)	
**Average CpG**			0.65
Median (IQR)	4.83 (2.83-6.67)	5.42 (2.67-12.67)	

*p p* value, *IQR* interquartile range

*P value is statistically significant at *p* ≤ 0.05

## Discussion

Cancer has been historically seen as a genetic disease [24]. Epigenetic modifications are more frequent than genetic changes, and occur early in carcinogenesis rendering them possible therapeutic targets [25]. In addition, they can represent a prognostic marker and predictor for drug resistance [26]. DNA hypermethylation of tumor suppressor genes and DNA repair genes with its subsequent silencing is one of the most common epigenetic modifications that lead to cancer progression [10, 27]. Like other cancers, ovarian cancer is associated with epigenetic changes [28].

Ovarian cancer is mainly a disease of post-menopausal women [29]. The incidence of ovarian cancer increases with age progress [30]. In our study, both age and menopausal status were matched between age group. Pregnancy/parity, [31] lactation, [32] tubal ligation, [29] and OCPs, [33] are all protective factors against ovarian cancer. In our study, the protective mechanism of pregnancy/parity and lactation was demonstrated. However, there was no difference regarding tubal ligation and OCPs due to the reduced access of subjects to contraception.

In our study, CA125 was done for all subjects. We found a statistically significant difference between the studied groups. Using a very near cutoff to the well-established 35 IU /ml, our figures for sensitivity and specificity were similar to what was found in the literature; 70-80 and 87% respectively [6, 8]. Similarly, RMI was calculated for all subjects with adnexal masses and sensitivity and specificity were calculated. Using 200 as the cutoff, our sensitivity (88%) was comparable to the reported sensitivity (85%) while the specificity was considerably lower (73.3% in comparison to 96.9%) [9]. However, CA125 and RMI are not suitable for screening asymptomatic women for ovarian cancer. Owing to the relatively low abundance of ovarian cancer and to avoid unnecessary laparotomies, a screening, a screening test should have a 99.6% sensitivity, 75% specificity and a positive predictive value (PPV) ≥10 [6, 34]. In fact, the current US and UK recommendations do not recommend using CA125 to screen women for ovarian cancer [35, 36].

In this study, we investigated the methylation status of *PCDH17* gene promoter. We found statistically significant hypermethylation in five out of the six CpG sites in the assay as well as in the mean CpG methylation. This result was in concordance with what was reported by Baranova and her colleagues [20]. One difference is that they investigated this using next-generation sequencing technology (NGS) which provides sequencing of the whole promoter. In our study, we used pyrosequencing technology which sequences short reads. *PCDH17* gene promoter has 6 CpG assays ready-made for the PyroMark® encompassing its length. In our study, we used the assay that flanks the part with the hypermethylated CpG sites described by Baranova and her colleagues. They also confirmed the hypermethylation silencing of gene expression by measuring *PCDH17* mRNA transcripts and found a negative correlation between both.

Another difference is that their study compared methylation between high-grade serous ovarian carcinoma to normal ovarian tissue [20]. To the contrary, our study included different types of epithelial ovarian cancer and their methylation was compared to a control group consisting of benign ovarian tumors and normal ovarian tissues. The inclusion of benign tumors in the control group was found to increase the diagnostic utility of the study and was found to be clinically useful given the classic presentation of pelvic/adnexal mass [37].

In addition, the same group recently investigated *PCDH17* gene methylation (with other genes namely *CDH13*, *HNF1B* and *GATA4*) in high grade serous ovarian carcinoma [21]. They reported a 100% specificity and an 88.5% sensitivity for the four-gene panel. For *PCDH17* alone, the specificity was 100% with a 60.7% sensitivity. This demonstrates the power of multiplexing. However, *PCDH17* alone had better sensitivity than the sensitivity of using the other genes alone (19.7, 50.8 and 31.2% respectively). This emphasizes the relative importance of *PCDH17* gene promoter hypermethylation in ovarian cancer diagnosis. Unlike their previous study, they investigated this gene panel using high-resolution melting analysis and methylation specific PCR. Similar to their previous study, they described a statistically significant decreased expression of *PCDH17* in hypermethylated samples.

Methylation levels and other epigenetic changes differs among different populations [25]. Methylation levels are affected by environmental factors, age and sex [38]. In our study, these confounding effects were considered to ensure the validity of the results. All our subjects were Egyptian females living in the same governorate and age was matched.

*PCDH17* gene promoter hypermethylation has been described to be found in different cancers other than ovarian cancer. For example, It was reported in in breast cancer [18], prostate cancer [19], and bladder cancer [39]. Furthermore, loss of *PCDH17* was described to favor metastasis in hepatocellular carcinoma [17]. More research on *PCDH17* in ovarian cancer is still needed to verify our findings as well as those of Baranova and her colleagues [20, 21].

The role of epigenetics in diagnosis of ovarian cancer is of paramount importance. Many gene hypermethylation changes have been reported [22, 40]. Identified DNA methylation markers serve as a potential for early diagnosis by investigating for their presence in cell-free DNA (cf-DNA) in serum [37, 41–43]. However, this was not assessed in our current study.

In addition to diagnosis, various histopathogic types of epithelial ovarian cancer have been classified according to their DNA methylation patterns [44, 45]. Drugs addressing epigenetic targets have been involved in clinical trials, [14] and ovarian cancer has been included in these trials [11]. Methylation characteristics can predict resistance to chemotherapy and confers poor prognosis in high grade serous ovarian cancer [46, 47].

To the best of our knowledge, this study is the second trial to assess *PCDH17* gene promoter hypermethylation in ovarian cancer after the fore-mentioned studies of Baranova and colleagues as well the first one using pyrosequencing technology. Pyrosequencing is a reproducible, and easy-to-use technique. It is considered the gold-standard for methylation detection as it allows the detection of methylation of individual CpG sites [48]. Hence, it can be used for validation of NGS data. Regarding cases, our study is the first in Egyptian population. Furthermore, it included benign ovarian tumors in the control group to ensure the usefulness of the results as the nature of ovarian masses is always a clinical interest. One limitation of this study is the relatively small size of the sample enrolled. Due to the small size, we have not been able to assess DNA methylation pattern in different tumor subgroups. The results of our study needs to be verified using larger sample cohorts in addition to exploring other CpG sites of the *PCDH17* gene promoter. Since this study was limited to Egyptian females, it needs to be replicated among other populations and ethnicities. In addition to this, using the *PCDH17* methylation pattern in cf-DNA would increase our understanding of the usefulness of *PCDH17* in ovarian cancer diagnosis and screening.

## Conclusions

We conclude that *PCDH17* gene promoter hypermethylation has a potential role in diagnosis of epithelial ovarian cancer.

## Data Availability

This study used pyrosequencing of a limited and well described promoter area with no new discovery to be deposited in databases. Pyrosequencing data are available from the corresponding author on reasonable request.
